# On rational bounds for the gamma function

**DOI:** 10.1186/s13660-017-1484-y

**Published:** 2017-09-08

**Authors:** Zhen-Hang Yang, Wei-Mao Qian, Yu-Ming Chu, Wen Zhang

**Affiliations:** 1 Department of Mathematics, Huzhou University, Huzhou, 313000 China; 2 Customer Service Center, State Grid Zhejiang Electric Power Research Institute, Hangzhou, 310009 China; 3 School of Distance Education, Huzhou Broadcast and TV University, Huzhou, 313000 China; 4 Friedman Brain Institute, Icahn School of Medicine at Mount Sinai, New York, 10029 USA

**Keywords:** 41A60, 33B15, 26D07, gamma function, psi function, rational bound, completely monotonic function

## Abstract

In the article, we prove that the double inequality

$$ \frac{x^{2}+p_{0}}{x+p_{0}}< \Gamma(x+1)< \frac{x^{2}+9/5}{x+9/5} $$

holds for all $x\in(0, 1)$, we present the best possible constants *λ* and *μ* such that

$$ \frac{\lambda(x^{2}+9/5)}{x+9/5}\leq\Gamma(x+1)\leq\frac{\mu (x^{2}+p_{0})}{x+p_{0}} $$

for all $x\in(0, 1)$, and we find the value of $x^{\ast}$ in the interval $(0, 1)$ such that $\Gamma(x+1)>(x^{2}+1/\gamma)/(x+1/\gamma)$ for $x\in(0, x^{\ast})$ and $\Gamma(x+1)<(x^{2}+1/\gamma)/(x+1/\gamma )$ for $x\in(x^{\ast}, 1)$, where $\Gamma(x)$ is the classical gamma function, $\gamma=\lim_{n\rightarrow\infty}(\sum_{k=1}^{n}1/k-\log n)=0.577\ldots$ is Euler-Mascheroni constant and $p_{0}=\gamma/(1-\gamma )=1.365\ldots$ .

## Introduction

For $x>0$, the classical Euler gamma function $\Gamma(x)$ and its logarithmic derivative, the so-called psi function $\psi(x)$ [1] are defined by

$$ \Gamma(x)= \int_{0}^{\infty}t^{x-1}e^{-t}\,dt, \qquad\psi(x)=\frac{\Gamma ^{\prime}(x)}{\Gamma(x)}, $$

respectively.

A real-valued function *f* is said to be completely monotonic [2] on an interval *I* if *f* has derivatives of all orders on *I* and $(-1)^{n}f^{(n)}(x)\geq0$ for all $n\geq0$ and $x\in I$. The well-known Bernstein theorem [3] states that a function *f* on $[0,\infty)$ is completely monotonic if and only if there exists a bounded and non-decreasing function $\omega(t)$ such that $f(x)=\int_{0}^{\infty }e^{-xt}\,d\omega(t)$ converges for all $x\in[0, \infty)$.

Recently, the gamma function have attracted the attention of many researchers. In particular, many remarkable inequalities and properties for $\Gamma(x)$ can be found in the literature [4–14].

Due to $\Gamma(x+1)=x\Gamma(x)$ and $\Gamma(n+1)=n!$, we will only need to focus our attention on $\Gamma(x+1)$ with $x\in(0, 1)$. Gautschi [15] proved that the double inequality

1.1 $$ n^{1-s}< \frac{\Gamma(n+1)}{\Gamma(n+s)}< e^{(1-s)\psi(n+1)} $$

holds for all $s\in(0, 1)$ and $n\in\mathbb{N}$.

Inequality (1.1) was generalized and improved by Kershaw [16] as follows:

$$ \biggl(x+\frac{s}{2} \biggr)^{1-s}< \frac{\Gamma(x+1)}{\Gamma (x+s)}< e^{(1-s)\psi[x+(1+s)/2]} $$

for all $x>0$ and $s\in(0, 1)$.

Elezović, Giordano and Pečarić [17] established the double inequality

1.2 $$ \biggl(\frac{1}{2}+\sqrt{\frac{1}{4}+x} \biggr)^{1-x}x^{x}< \Gamma (x+1)< 2^{1-x}x^{x} $$

for the gamma function being valid for all $x\in(0, 1)$, and asked for ‘other bounds for the gamma function in terms of elementary functions’.

Ivády [18] provided the bounds for gamma function in terms of very simple rational functions as follows:

1.3 $$ \frac{x^{2}+1}{x+1}< \Gamma(x+1)< \frac{x^{2}+2}{x+2} $$

for all $x\in(0, 1)$. Inequality (1.3) can be regarded as a simple estimation of the value of the gamma function.

In [19], Zhao, Guo and Qi proved that the function

$$ x\rightarrow Q(x)=\frac{\log\Gamma(x+1)}{\log(x^{2}+1)-\log(x+1)} $$

is strictly increasing on $(0, 1)$. The monotonicity of $Q(x)$ on the interval $(0, 1)$ and the facts that $Q(0^{+})=\gamma$ and $Q(1^{-})=2(1-\gamma)$ lead to the conclusion that

1.4 $$ \biggl(\frac{x^{2}+1}{x+1} \biggr)^{2(1-\gamma)}< \Gamma(x+1)< \biggl( \frac {x^{2}+1}{x+1} \biggr)^{\gamma} $$

for all $x\in(0, 1)$, where $\gamma=\lim_{n\rightarrow\infty}(\sum_{k=1}^{n}1/k-\log n)=0.577\ldots$ is the Euler-Mascheroni constant.

Let

1.5 $$\begin{aligned}& L_{1}(x)=\frac{x^{2}+1}{x+1}, \qquad L_{2}(x)= \biggl( \frac {x^{2}+1}{x+1} \biggr)^{2(1-\gamma)}, \end{aligned}$$

1.6 $$\begin{aligned}& U_{1}(x)=\frac{x^{2}+2}{x+2}, \qquad U_{2}(x)= \biggl( \frac {x^{2}+1}{x+1} \biggr)^{\gamma}. \end{aligned}$$

Then we clearly see that

1.7 $$ L_{1}(x)< L_{2}(x) $$

for all $x\in(0, 1)$, and numerical computations show that

1.8 $$ \begin{gathered} U_{1}(1/4)=0.916\ldots>U_{2}(1/4)=0.910\ldots, \\ U_{1}(1/8)=0.948\ldots>U_{2}(1/8)=0.942\ldots. \end{gathered}$$

Motivated by (1.3)-(1.8), it is natural to ask what the better parameters *p* and *q* on the interval $(1, 2)$ are such that the double inequality

$$ \frac{x^{2}+p}{x+p}< \Gamma(x+1)< \frac{x^{2}+q}{x+q} $$

holds for all $x\in(0, 1)$. The main purpose of the article is to deal with this questions. Some complicated computations are carried out using the Mathematica computer algebra system.

## Lemmas

In order to establish our main results we need several lemmas, which we present in this section.

### Lemma 2.1

See [20, Theorem 1.25]

*Let* $-\infty< a< b<\infty$, $f,g:[a,b]\rightarrow{\mathbb{R}}$ *be continuous on* $[a,b]$ *and differentiable on* $(a,b)$, *and* $g'(x)\neq0$ *on* $(a,b)$. *If* $f^{\prime}(x)/g^{\prime}(x)$ *is increasing* (*decreasing*) *on* $(a,b)$, *then so are the functions*

$$ \frac{f(x)-f(a)}{g(x)-g(a)}, \quad\quad\frac{f(x)-f(b)}{g(x)-g(b)}. $$

*If* $f^{\prime}(x)/g^{\prime}(x)$ *is strictly monotone*, *then the monotonicity in the conclusion is also strict*.

### Lemma 2.2

See [21, Lemma 7]

*Let* $n\in\mathbb{N}$ *and* $m\in\mathbb{N}\cup\{0\}$ *with* $n>m$, $a_{i}\geq0$ *for all* $0\leq i\leq n$, $a_{n}a_{m}>0$ *and*

$$ P_{n}(t)=-\sum_{i=0}^{m}a_{i}t^{i}+ \sum_{i=m+1}^{n}a_{i}t^{i}. $$

*Then there exists* $t_{0}\in(0, \infty)$ *such that* $P_{n}(t_{0})=0$, $P_{n}(t)<0$ *for* $t\in(0, t_{0})$ *and* $P_{n}(t)>0$ *for* $t\in(t_{0}, \infty)$.

### Lemma 2.3

See [22, Corollary 3.1]

*The inequality*

$$ \psi(x+1)< \frac{1}{92}\log \biggl(x^{2}+x+\frac{4}{3} \biggr)+\frac {45}{92}\log \biggl(x^{2}+x+\frac{14}{45} \biggr) $$

*holds for all* $x>0$.

### Lemma 2.4

See [23, Corollary 3.3(ii)]

*The double inequality*

$$\begin{aligned} \frac{ (x+\frac{1}{2} ) (x^{2}+x+\frac{23}{21} )}{x^{4}+2x^{3}+\frac{17}{7}x^{2}+\frac{10}{7}x+\frac{12}{35}}&< \psi ^{\prime}(x+1) \\ &< \frac{ (x+\frac{1}{2} ) (x^{2}+x+\frac{\pi^{2}}{15(\pi ^{2}-9)} )}{ x^{4}+2x^{3}+\frac{7\pi^{2}-60}{5(\pi^{2}-9)}x^{2}+\frac{2\pi ^{2}-15}{5(\pi^{2}-9)}x+\frac{1}{5(\pi^{2}-9)}} \end{aligned}$$

*holds for all* $x>0$.

### Lemma 2.5

*The inequalities*

2.1 $$\begin{aligned}& \frac{1}{x+\frac{1}{2}}-\frac{1}{12 (x+\frac{1}{2} )^{3}}\leq \psi^{\prime}(x+1)\leq \frac{1}{x+\frac{1}{2}}, \end{aligned}$$

2.2 $$\begin{aligned}& -\frac{1}{ (x+\frac{1}{2} )^{2}}\leq\psi^{\prime\prime }(x+1)\leq-\frac{1}{ (x+\frac{1}{2} )^{2}} + \frac{1}{4 (x+\frac{1}{2} )^{4}}, \end{aligned}$$

2.3 $$\begin{aligned}& \frac{2}{ (x+\frac{1}{2} )^{3}}-\frac{1}{ (x+\frac {1}{2} )^{5}}\leq\psi^{\prime\prime\prime}(x+1) \leq \frac{2}{ (x+\frac{1}{2} )^{3}} \end{aligned}$$

*hold for all* $x>-1/2$.

### Proof

Let $x>-1/2$, and $R_{1}(x)$ and $R_{2}(x)$ be defined by

2.4 $$\begin{aligned}& R_{1}(x)=\psi(x+1)-\log \biggl(x+\frac{1}{2} \biggr), \end{aligned}$$

2.5 $$\begin{aligned}& R_{2}(x)=-\psi(x+1)+\log \biggl(x+\frac{1}{2} \biggr)+ \frac{1}{24 (x+\frac{1}{2} )^{2}}, \end{aligned}$$

respectively. Then making use of the well-known formulas

$$ \psi(x)= \int_{0}^{\infty} \biggl(\frac{e^{-t}}{t}- \frac {e^{-xt}}{1-e^{-t}} \biggr)\,dt, \qquad\log x= \int_{0}^{\infty}\frac {e^{-t}-e^{-xt}}{t}\,dt $$

we get

2.6 $$\begin{aligned}& \begin{aligned} R_{1}(x)&= \int_{0}^{\infty} \biggl(\frac{e^{-t}}{t}- \frac {e^{-(x+1)t}}{1-e^{-t}} \biggr)\,dt- \int_{0}^{\infty}\frac {e^{-t}-e^{-(x+1/2)t}}{t}\,dt \\ &= \int_{0}^{\infty} \biggl(\frac{1}{t}- \frac{e^{-t/2}}{1-e^{-t}} \biggr)e^{-(x+1/2)t}\,dt \\ &= \int_{0}^{\infty} \biggl(\frac{1}{t}- \frac{1}{2\sinh(t/2)} \biggr)e^{-(x+1/2)t}\,dt \\ &= \int_{0}^{\infty}\frac{\sinh(t/2)-t/2}{t\sinh(t/2)}e^{-(x+1/2)t}\,dt, \end{aligned} \end{aligned}$$

2.7 $$\begin{aligned}& \begin{aligned}[b] R_{2}(x)&=- \int_{0}^{\infty} \biggl(\frac{1}{t}- \frac{1}{2\sinh (t/2)} \biggr)e^{-(x+1/2)t}\,dt+\frac{1}{24} \int_{0}^{\infty} te^{-(x+1/2)t}\,dt \\ &= \int_{0}^{\infty} \biggl(\frac{(t^{2}-24)\sinh(t/2)+12t}{24t\sinh (t/2)} \biggr)e^{-(x+1/2)t}\,dt, \end{aligned} \end{aligned}$$

where $\sinh(t)=(e^{t}-e^{-t})/2$ is the hyperbolic sine function.

Note that

2.8 $$\begin{aligned}& 6t\sinh(t/2)\frac{(t^{2}-24)\sinh(t/2)+12t}{24t\sinh(t/2)}=\sum_{n=3}^{\infty} \frac{2(n-2)(2n+1)}{(2n-3)!} \biggl(\frac{t}{2} \biggr)^{2n-1}>0, \end{aligned}$$

2.9 $$\begin{aligned}& \sinh(t/2)-\frac{t}{2}>0 \end{aligned}$$

for $t>0$.

It follows from (2.6)-(2.9) and the Bernstein theorem for complete monotonicity property that the two functions $R_{1}(x)$ and $R_{2}(x)$ are completely monotonic on the interval $(-1/2, \infty)$.

Therefore, Lemma 2.5 follows easily from (2.4), (2.5) and the complete monotonicity of $R_{1}(x)$ and $R_{2}(x)$ on the interval $(-1/2, \infty )$ together with the facts that

$$ \biggl[\log \biggl(x+\frac{1}{2} \biggr) \biggr]^{(n)}= \frac {(-1)^{n-1}(n-1)!}{ (x+\frac{1}{2} )^{n}},\qquad \biggl[\frac{1}{ (x+\frac{1}{2} )^{2}} \biggr]^{(n)}= \frac {(-1)^{n}(n+1)!}{ (x+\frac{1}{2} )^{n+2}}. $$

 □

### Lemma 2.6

*The double inequality*

2.10 $$ \biggl(\frac{x^{2}+1}{x+1} \biggr)^{2/(q+1)}< \frac{x^{2}+q}{x+q}< \biggl( \frac{x^{2}+1}{x+1} \biggr)^{1/q} $$

*holds for all* $x\in(0, 1)$ *and* $q>1$.

### Proof

Let $x\in(0, 1)$, $q>1$, and $H_{1}(x)$ and $H_{2}(x)$ be defined by

2.11 $$ H_{1}(x)=\log\bigl(x^{2}+q\bigr)-\log(x+q), \qquad H_{2}(x)=\log\bigl(x^{2}+1\bigr)-\log(x+1), $$

respectively. Then simple computations lead to

2.12 $$\begin{aligned}& \lim_{x\rightarrow0^{+}}\frac{H_{1}(x)}{H_{2}(x)}=\frac{1}{q}, \qquad \lim _{x\rightarrow1^{-}}\frac{H_{1}(x)}{H_{2}(x)}=\frac{2}{q+1}, \end{aligned}$$

2.13 $$\begin{aligned}& H_{1}\bigl(0^{+}\bigr)=H_{2} \bigl(0^{+}\bigr)=0, \end{aligned}$$

2.14 $$\begin{aligned}& H_{1}\bigl(1^{-}\bigr)=H_{2} \bigl(1^{-}\bigr)=0, \end{aligned}$$

2.15 $$\begin{aligned}& \frac{H_{1}^{\prime}(x)}{H_{2}^{\prime}(x)}=\frac {(x+1)(x^{2}+1)(x^{2}+2qx-q)}{(x+q)(x^{2}+q)(x^{2}+2x-1)}, \\& \biggl[\frac{H_{1}^{\prime}(x)}{H_{2}^{\prime}(x)} \biggr]^{\prime}=\frac {(q-1)\triangle(x,q)}{(x+q)^{2}(x^{2}+q)^{2}(x^{2}+2x-1)^{2}}, \end{aligned}$$

where

2.16 $$ \begin{aligned}[b] \triangle (x,q)={}&\bigl[8x^{5}+4x^{4}+(1-x)^{3} \bigl(4x^{2}+3x+1\bigr)\bigr]q^{2}+4x^{2} \bigl[2x^{4}+4x^{3}+(1-x)^{2}\bigr]q \\ &-x^{4}\bigl(x^{4}-4x^{3}-6x^{2}-4x+1 \bigr) \\ >{}&\triangle (x,1)\\ ={}&(1-x)x^{7}+3x^{7}+10x^{6}+ \bigl(2x^{3}-1\bigr)^{2}+24x^{5} +8x^{4}+2x^{2}(2x-1)^{2}\\ >{}&0. \end{aligned}$$

From (2.15) and (2.16) we clearly see that $H_{1}^{\prime }(x)/H_{2}^{\prime}(x)$ is strictly increasing on $(0, \sqrt{2}-1)\cup (\sqrt{2}-1, 1)$. We assert that the function $H_{1}(x)/H_{2}(x)$ is strictly increasing on $(0, 1)$. Indeed, if $x\in(0, \sqrt{2}-1)$, then $H_{2}^{\prime}(x)\neq0$, and Lemma 2.1 and (2.13) together with the monotonicity of $H_{1}^{\prime}(x)/H_{2}^{\prime}(x)$ on $(0, \sqrt {2}-1)$ lead to the conclusion that $H_{1}(x)/H_{2}(x)$ is strictly increasing on $(0, \sqrt{2}-1)$; if $x\in(\sqrt{2}-1, 1)$, then $H_{2}^{\prime}(x)\neq0$, and Lemma 2.1 and (2.14) together with the monotonicity of $H_{1}^{\prime}(x)/H_{2}^{\prime}(x)$ on $(\sqrt {2}-1, 1)$ lead to the conclusion that $H_{1}(x)/H_{2}(x)$ is strictly increasing on $(\sqrt{2}-1, 1)$.

Therefore, Lemma 2.6 follows easily from (2.11) and (2.12) together with the monotonicity of the function $H_{1}(x)/H_{2}(x)$ on $(0, 1)$. □

Let $p>0$, $x\in(0, 1)$, and $f(p; x)$, $f_{1}(p; x)$, $f_{2}(p; x)$ and $f_{3}(p; x)$ be defined by

2.17 $$\begin{aligned}& f(p, x)=\log\Gamma(x+1)-\log \biggl(\frac{x^{2}+p}{x+p} \biggr), \end{aligned}$$

2.18 $$\begin{aligned}& f_{1}(p, x)=\frac{\partial f(p, x)}{\partial x}=\psi(x+1)-\frac {2x}{x^{2}+p}+ \frac{1}{x+p}, \end{aligned}$$

2.19 $$\begin{aligned}& f_{2}(p, x)=\frac{\partial^{2} f(p, x)}{\partial x^{2}}=\psi^{\prime }(x+1)+ \frac{4x^{2}}{(x^{2}+p)^{2}}-\frac{2}{x^{2}+p}-\frac{1}{(x+p)^{2}}, \end{aligned}$$

2.20 $$\begin{aligned}& f_{3}(p, x)=\frac{\partial^{3} f(p, x)}{\partial x^{3}}=\psi^{\prime \prime}(x+1)- \frac{16x^{3}}{(x^{2}+p)^{3}}+\frac {12x}{(x^{2}+p)^{2}}+\frac{2}{(x+p)^{3}}. \end{aligned}$$

### Lemma 2.7

*Let* $f_{2}(p, x)$ *be defined by* (2.19). *Then*

2.21 $$ f_{2}(p, 1/3)< 0 $$

*for* $p\in[8/5, 9/5]$.

### Proof

From (2.19) and the second inequality in Lemma 2.4 we have

2.22 $$ \begin{aligned}[b] f_{2}(p, 1/3)={}& \biggl[\psi^{\prime}(x+1)+\frac {4x^{2}}{(x^{2}+p)^{2}}- \frac{2}{x^{2}+p}-\frac{1}{(x+p)^{2}} \biggr]_{x=1/3} \\ < {}& \biggl[\frac{ (x+\frac{1}{2} ) (x^{2}+x+\frac{\pi ^{2}}{15(\pi^{2}-9)} )}{ x^{4}+2x^{3}+\frac{7\pi^{2}-60}{5(\pi^{2}-9)}x^{2}+\frac{2\pi ^{2}-15}{5(\pi^{2}-9)}x+\frac{1}{5(\pi^{2}-9)}} \biggr]_{x=1/3} \\ &+ \biggl[\frac{4x^{2}}{(x^{2}+p)^{2}}-\frac{2}{x^{2}+p}-\frac {1}{(x+p)^{2}} \biggr]_{x=1/3} \\ ={}&\frac{15(23\pi^{2}-180)}{2(152\pi^{2}-1\mbox{,}179)}-\frac {9(162p^{3}+171p^{2}+24p-1)}{(3p+1)^{2}(9p+1)^{2}}. \end{aligned}$$

Elaborated computations lead to

2.23 $$ \begin{aligned}[b] & \biggl(\frac{15(23\pi^{2}-180)}{2(152\pi^{2}-1\mbox{,}179)}-\frac {9(162p^{3}+171p^{2}+24p-1)}{ (3p+1)^{2}(9p+1)^{2}} \biggr)^{\prime} \\ &\quad=\frac{54(729p^{4}+1\mbox{,}215p^{3}+243p^{2}-27p-8)}{(3p+1)^{3}(9p+1)^{3}}>0 \end{aligned}$$

for $p\in[8/5, 9/5]$.

From (2.22) and (2.23) we get

$$\begin{aligned} f_{2}(p, 1/3)&< \biggl[\frac{15(23\pi^{2}-180)}{2(152\pi^{2}-1\mbox{,}179)}-\frac {9(162p^{3}+171p^{2}+24p-1)}{(3p+1)^{2}(9p+1)^{2}} \biggr]_{p=9/5} \\ &=\frac{15(23\pi^{2}-180)}{2(152\pi^{2}-1\mbox{,}179)}-\frac {2\mbox{,}167\mbox{,}065}{1\mbox{,}893\mbox{,}376}=-0.047\ldots< 0. \end{aligned}$$

 □

### Lemma 2.8

*Let* $f_{2}(p, x)$ *be defined by* (2.19). *Then*

2.24 $$ f_{2}(9/5, 7/50)>0. $$

### Proof

From (2.19) and the first inequality in Lemma 2.4 we have

$$\begin{aligned} f_{2}(9/5, 7/50)&= \biggl[\psi^{\prime}(x+1)+\frac {4x^{2}}{(x^{2}+p)^{2}}- \frac{2}{x^{2}+p}-\frac{1}{(x+p)^{2}} \biggr]_{p=9/5, x=7/50} \\ &> \biggl[\frac{ (x+\frac{1}{2} ) (x^{2}+x+\frac {23}{21} )}{x^{4}+2x^{3}+\frac{17}{7}x^{2} +\frac{10}{7}x+\frac{12}{35}}+\frac{4x^{2}}{(x^{2}+p)^{2}}-\frac {2}{x^{2}+p}- \frac{1}{(x+p)^{2}} \biggr]_{p=9/5, x=7/50} \\ &=\frac{84\mbox{,}826\mbox{,}873\mbox{,}256\mbox{,}410\mbox{,}100}{15\mbox{,}239\mbox{,}152\mbox{,}138\mbox{,}614\mbox{,}823\mbox{,}989}>0. \end{aligned}$$

 □

### Lemma 2.9

*Let* $f_{1}(p, x)$ *be defined by* (2.18). *Then*

$$ f_{1}(9/5, x)< 0 $$

*for* $x\in(7/50, 1/3)$.

### Proof

It follows from Lemma 2.3 and (2.18) that

2.25 $$ \begin{aligned}[b] f_{1}(9/5, x)< \frac{1}{92}\log \biggl(x^{2}+x+ \frac{4}{3} \biggr)+\frac {45}{92}\log \biggl(x^{2}+x+ \frac{14}{45} \biggr) -\frac{2x}{x^{2}+9/5}+\frac{1}{x+9/5}. \end{aligned}$$

Elaborated computations lead to

2.26 $$ \begin{aligned}[b] & \biggl[\frac{1}{92}\log \biggl(x^{2}+x+\frac{4}{3} \biggr)+\frac {45}{92}\log \biggl(x^{2}+x+\frac{14}{45} \biggr) -\frac{2x}{x^{2}+9/5}+\frac{1}{x+9/5} \biggr]^{\prime} \\ &\quad=\frac{h(x)}{2(5x+9)^{2}(5x^{2}+9)^{2}(3x^{2}+3x+4)(45x^{2}+45x+14)}, \end{aligned}$$

where

2.27 $$\begin{aligned}& \begin{aligned} h(x)={}&168\mbox{,}750x^{9}+1\mbox{,}029\mbox{,}375x^{8}+3\mbox{,}923\mbox{,}625x^{7}+7\mbox{,}884\mbox{,}000x^{6}+9\mbox{,}344\mbox{,}775x^{5} \\ &+5\mbox{,}316\mbox{,}100x^{4}+203\mbox{,}355x^{3}-2\mbox{,}426\mbox{,}940x^{2}-544\mbox{,}401x+118\mbox{,}017, \end{aligned} \\& h(7/50)=-\frac{406\mbox{,}357\mbox{,}216\mbox{,}255\mbox{,}013}{156\mbox{,}250\mbox{,}000\mbox{,}000}< 0, \end{aligned}$$

2.28 $$\begin{aligned}& \begin{aligned}[b] h^{\prime }(x)={}&1\mbox{,}518\mbox{,}750x^{8}+8\mbox{,}235\mbox{,}000x^{7}+27\mbox{,}465\mbox{,}375x^{6}+47\mbox{,}304\mbox{,}000x^{5}+46\mbox{,}723\mbox{,}875x^{4} \\ &+21\mbox{,}264\mbox{,}400x^{3}+610\mbox{,}065x^{2}-4\mbox{,}853\mbox{,}880x-544\mbox{,}401, \end{aligned} \end{aligned}$$

2.29 $$\begin{aligned}& h^{\prime}(1/3)=-\frac{120\mbox{,}000\mbox{,}368}{243}< 0. \end{aligned}$$

From Lemma 2.2, (2.28) and (2.29) we know that $h(x)$ is strictly decreasing on $(7/50, 1/3)$, then (2.27) leads to the conclusion that $h(x)<0$ for $x\in(7/50, 1/3)$.

Therefore,

$$\begin{aligned} f_{1}(9/5,x)< {}& \biggl[\frac{1}{92}\log \biggl(x^{2}+x+ \frac{4}{3} \biggr)+\frac{45}{92}\log \biggl(x^{2}+x+ \frac{14}{45} \biggr) \\ &-\frac{2x}{x^{2}+9/5}+\frac{1}{x+9/5} \biggr]_{x=7/50} \\ ={}&\frac{1}{92}\log\frac{11\mbox{,}197}{7\mbox{,}500}+\frac{45}{92}\log \frac {10\mbox{,}591}{22\mbox{,}500}+\frac{159\mbox{,}550}{441\mbox{,}253} =-0.0026\ldots< 0 \end{aligned}$$

for $x\in(7/50, 1/3)$ follows from (2.25) and (2.26) together with $h(x)<0$ for $x\in (7/50, 1/3)$. □

### Lemma 2.10

*Let* $p\in[3/2, 2]$ *and* $f_{3}(p, x)$ *be defined by* (2.20). *Then there exists* $\eta(p)\in(0, 1)$ *such that* $f_{3}(p, x)<0$ *for* $x\in (0, \eta(p))$ *and* $f_{3}(p, x)>0$ *for* $x\in(\eta(p), 1)$.

### Proof

Let

2.30 $$ g(p,x)=\bigl(x^{2}+p\bigr)^{3}f_{3}(p, x)= \bigl(x^{2}+p\bigr)^{3}\psi^{\prime\prime }(x+1)+ \frac{2(x^{2}+p)^{3}}{(x+p)^{3}}-4x^{3}+12px. $$

Then simple computations lead to

2.31 $$\begin{aligned}& g\bigl(p,0^{+}\bigr)=p^{3}\psi^{\prime\prime}(1)+2, \qquad g\bigl(p,1^{-}\bigr)=(p+1)^{3}\psi ^{\prime\prime}(2)+12p-2, \end{aligned}$$

2.32 $$\begin{aligned}& \begin{aligned}[b] \frac{\partial g(p,x)}{\partial x}={}&\bigl(x^{2}+p\bigr)^{3} \psi^{\prime\prime \prime}(x+1)+6x\bigl(x^{2}+p\bigr)^{2} \psi^{\prime\prime}(x+1) \\ &-\frac{6(x^{2}+p)^{3}}{(x+p)^{4}}+\frac {12x(x^{2}+p)^{2}}{(x+p)^{3}}-12x^{2}+12p. \end{aligned} \end{aligned}$$

It follows from the first inequalities in (2.2) and (2.3) together with the identity $\psi^{(n)}(x+1)=\psi^{(n)}(x)+(-1)^{n}n!/x^{n+1}$ that

2.33 $$\begin{aligned}& \psi^{\prime\prime}(x+1)=\psi^{\prime\prime}(x+2)-\frac{2}{(x+1)^{3}} \geq- \frac{1}{(x+3/2)^{2}}-\frac{2}{(x+1)^{3}}, \end{aligned}$$

2.34 $$\begin{aligned}& \psi^{\prime\prime\prime}(x+1)=\psi^{\prime\prime\prime}(x+2)+\frac {6}{(x+1)^{4}} \geq \frac{2}{(x+3/2)^{3}}-\frac{1}{(x+3/2)^{5}}+\frac{6}{(x+1)^{4}}. \end{aligned}$$

From (2.32)-(2.34) we have

2.35 $$ \begin{aligned}[b] \frac{\partial g(p,x)}{\partial x}\geq{}&\bigl(x^{2}+p\bigr)^{3} \biggl[ \frac {2}{(x+3/2)^{3}}-\frac{1}{(x+3/2)^{5}}+\frac{6}{(x+1)^{4}} \biggr] \\ &+6x\bigl(x^{2}+p\bigr)^{2} \biggl[-\frac{1}{(x+3/2)^{2}}- \frac{2}{(x+1)^{3}} \biggr] \\ &-\frac{6(x^{2}+p)^{3}}{(x+p)^{4}}+\frac {12x(x^{2}+p)^{2}}{(x+p)^{3}}-12x^{2}+12p \\ ={}&\frac{2g_{1}(p, x)}{(2x+3)^{5}(x+1)^{4}(x+p)^{4}}, \end{aligned}$$

where

2.36 $$ g_{1}(p, x)=\sum_{k=0}^{6}b_{k}x^{k}- \sum_{k=7}^{16}b_{k}x^{k} $$

with

2.37 $$\begin{aligned}& b_{0}=\bigl(785p^{4}+1\mbox{,}458p^{2}-729 \bigr)p^{3}, \\& b_{1}=2\bigl(1\mbox{,}375p^{4}+679p^{3}+5\mbox{,}346p^{2}+2\mbox{,}916p-1\mbox{,}944 \bigr)p^{3}, \\& b_{2}=\bigl(3\mbox{,}992p^{5}+5\mbox{,}093p^{4}+32\mbox{,}250p^{3}+41\mbox{,}310p^{2}+2\mbox{,}106p-729 \bigr)p^{2}, \\& b_{3}=2\bigl(1\mbox{,}544p^{5}+3\mbox{,}955p^{4}+27\mbox{,}270p^{3}+60\mbox{,}214p^{2}+30\mbox{,}186p+1\mbox{,}701 \bigr)p^{2}, \\& b_{4}=\bigl(1\mbox{,}352p^{6}+6\mbox{,}520p^{5}+56\mbox{,}049p^{4}+187\mbox{,}562p^{3}+184\mbox{,}880p^{2}+38\mbox{,}070p+2\mbox{,}187 \bigr)p, \\& b_{5}=2\bigl(160p^{6}+1\mbox{,}510p^{5}+16\mbox{,}743p^{4}+78\mbox{,}961p^{3}+128\mbox{,}815p^{2}+54\mbox{,}729p+5\mbox{,}832 \bigr)p, \\& b_{6}=32p^{7}+752p^{6}+8\mbox{,}088p^{5}+44\mbox{,}529p^{4}+135\mbox{,}746p^{3}+123\mbox{,}972p^{2}+19\mbox{,}926p-729, \\& b_{7}=-80p^{6}+3\mbox{,}000p^{5}+47\mbox{,}018p^{4}+106\mbox{,}872p^{3}+27\mbox{,}156p^{2}+9\mbox{,}720p+5\mbox{,}346, \\& b_{8}=2\mbox{,}904p^{5}+60\mbox{,}104p^{4}+245\mbox{,}744p^{3}+259\mbox{,}818p^{2}+99\mbox{,}246p+17\mbox{,}334, \\& b_{9}=864p^{5}+31\mbox{,}572p^{4}+204\mbox{,}464p^{3}+360\mbox{,}816p^{2}+195\mbox{,}790p+34\mbox{,}398, \\& b_{10}=96p^{5}+8\mbox{,}896p^{4}+96\mbox{,}576p^{3}+272\mbox{,}304p^{2}+216\mbox{,}076p+46\mbox{,}726, \\& b_{11}=1\mbox{,}264p^{4}+26\mbox{,}944p^{3}+126\mbox{,}120p^{2}+151\mbox{,}960p+44\mbox{,}884, \\& b_{12}=64p^{4}+4\mbox{,}096p^{3}+35\mbox{,}712p^{2}+69\mbox{,}752p+30\mbox{,}400, \\& b_{13}=256p^{3}+5\mbox{,}664p^{2}+20\mbox{,}320p+14\mbox{,}180, \\& b_{14}=384p^{2}+3\mbox{,}424p+4\mbox{,}336, \\& b_{15}=256p+784, \\& b_{16}=16, \\& \begin{aligned}[b] g_{1}(p,1)={}&{-}199\mbox{,}181-732\mbox{,}767p-813\mbox{,}801p^{2}-48\mbox{,}835p^{3}+408\mbox{,}665p^{4} \\ &+189\mbox{,}699p^{5}+24\mbox{,}733p^{6}+12\mbox{,}319p^{7}, \end{aligned} \end{aligned}$$

2.38 $$\begin{aligned}& g_{1}(3/2,1)=\frac{90\mbox{,}546\mbox{,}875}{128}>0. \end{aligned}$$

From Lemma 2.2, (2.37) and (2.38) we clearly see that

2.39 $$ g_{1}(p,1)>0 $$

for $p\in[3/2, 1]$.

Making use of Lemma 2.2 again, and (2.36) and (2.39) together with the facts that ${b_{k}>0}$ for $p\in[3/2, 1]$ and $k=0, 1, 2, \ldots, 16$ we know that $g_{1}(p, x)>0$ for $p\in[3/2, 1]$ and $x\in(0, 1)$. Then inequality (2.35) leads to the conclusion that the function $x\rightarrow g(p, x)$ is strictly increasing on $(0, 1)$ for $p\in [3/2, 2]$.

From (2.2) and the identity $\psi^{(n)}(x)=\psi ^{(n)}(x+1)+(-1)^{n+1}n!/x^{n+1}$ we get

2.40 $$ \begin{aligned}[b] -\frac{1}{(x+1/2)^{2}}&\leq\psi^{\prime\prime}(x+1)=\psi^{\prime\prime }(x+2)- \frac{2}{(x+1)^{3}} \\ &\leq-\frac{1}{(x+3/2)^{2}}+\frac{1}{4(x+3/2)^{4}}-\frac{2}{(x+1)^{3}}. \end{aligned}$$

Taking $x=1$ in the first inequality of (2.40) and $x=0$ in the second inequality of (2.40), one has

2.41 $$ \psi^{\prime\prime}(2)\geq-\frac{4}{9}, \qquad\psi^{\prime\prime }(1)\leq- \frac{194}{81}. $$

It follows from (2.31) and (2.41) that

2.42 $$\begin{aligned}& g\bigl(p, 0^{+}\bigr)\leq-\frac{194}{81}\times \biggl( \frac{3}{2} \biggr)^{3}+2=-\frac{73}{12}< 0, \end{aligned}$$

2.43 $$\begin{aligned}& g\bigl(p, 1^{-}\bigr)\geq-\frac{4}{9}(p+1)^{3}+12p-2 \end{aligned}$$

for $p\in[3/2, 2]$.

Note that

2.44 $$ \biggl[-\frac{4}{9}(p+1)^{3}+12p-2 \biggr]^{\prime}= \frac{4}{3}(p+4) (2-p). $$

Inequality (2.43) and equation (2.44) imply that

2.45 $$ g\bigl(p, 1^{-}\bigr)\geq-\frac{4}{9} \biggl( \frac{3}{2}+1 \biggr)^{3}+12\times\frac {3}{2}-2= \frac{163}{18}>0 $$

for $p\in[3/2, 2]$.

Therefore, Lemma 2.10 follows easily from (2.30), (2.42), (2.45) and the monotonicity of the function $x\rightarrow g(p, x)$ on the interval $(0, 1)$. □

### Lemma 2.11

*Let* $p\in[8/5, 9/5]$ *and* $f_{2}(p, x)$ *be defined by* (2.19). *Then there exist* $\eta_{1}(p), \eta_{2}(p)\in(0, 1)$ *with* $\eta_{1}(p)<\eta _{2}(p)$ *such that* $f_{2}(p, x)>0$ *for* $x\in(0, \eta_{1}(p))\cup(\eta _{2}(p), 1)$ *and* $f_{2}(p, x)<0$ *for* $x\in(\eta_{1}(p), \eta_{2}(p))$.

### Proof

It follows from (2.19) that

2.46 $$\begin{aligned}& f_{2}\bigl(p, 0^{+}\bigr)=\frac{\pi^{2}}{6p^{2}} \biggl(p- \frac{\sqrt{6(\pi ^{2}+6)}+6}{\pi^{2}} \biggr) \biggl(p+\frac{\sqrt{6(\pi^{2}+6)}-6}{\pi^{2}} \biggr)>0, \end{aligned}$$

2.47 $$\begin{aligned}& f_{2}\bigl(p, 1^{-}\bigr)=\frac{(\pi^{2}-6)p^{2}+2(\pi^{2}-12)p+\pi^{2}}{6(p+1)^{2}}>0 \end{aligned}$$

for $p\in[8/5, 9/5]$.

From Lemma 2.10 and $[8/5, 9/5]\subset[3/2, 2]$ we know that there exists $\eta(p)\in(0, 1)$ such that the function $x\rightarrow f_{2}(p, x)$ is strictly decreasing on $(0, \eta(p))$ and strictly increasing on $(\eta(p), 1)$. Then Lemma 2.7 leads to the conclusion that

2.48 $$ f_{2}\bigl(p, \eta(p)\bigr)\leq f_{2}(p, 1/3)< 0. $$

Therefore, there exist $\eta_{1}(p)\in(0, \eta(p))$ and $\eta _{2}(p)\in(\eta(p), 1)$ such that $f_{2}(p, x)>0$ for $x\in(0, \eta_{1}(p))\cup(\eta_{2}(p), 1)$ and $f_{2}(p, x)<0$ for $x\in(\eta_{1}(p), \eta_{2}(p))$ follow from (2.46)-(2.48) and the piecewise monotonicity of the function $x\rightarrow f_{2}(p, x)$ on the interval $(0, 1)$. □

## Main results

### Theorem 3.1

*Let* $p>0$ *and* $p_{0}=\gamma/(1-\gamma)=1.365\ldots$ . *Then the inequality*

3.1 $$ \Gamma(x+1)>\frac{x^{2}+p}{x+p} $$

*holds for all* $x\in(0, 1)$ *if and only if* $p\leq p_{0}$, *and the inequality*

3.2 $$ \Gamma(x+1)\leq\frac{\mu(x^{2}+p_{0})}{x+p_{0}} $$

*holds for all* $x\in(0, 1)$ *if and only if* $\mu\geq\mu_{0}$, *where*

3.3 $$ \mu_{0}=\frac{(x_{0}+p_{0})\Gamma (x_{0}+1)}{(x^{2}_{0}+p_{0})}=1.027\ldots $$

*and* $x_{0}=0.346\ldots$ *is the unique solution of the equation*

3.4 $$ \psi(x+1)-\frac{2x}{x^{2}+p_{0}}+\frac{1}{x+p_{0}}=0 $$

*on the interval* $(0, 1)$.

### Proof

If inequality (3.1) holds for all $x\in(0, 1)$, then $p\leq p_{0}$ follows easily from

$$ \lim_{x\rightarrow1^{-}}\frac{\log\Gamma(x+1)-\log (\frac {x^{2}+p}{x+p} )}{1-x}=-\psi(2)+\frac{1}{1+p}= \gamma-\frac {p}{1+p}\geq0. $$

Next, we prove that inequality (3.1) holds for all $x\in(0, 1)$ and $p=p_{0}$ and (3.2) holds for all $x\in(0, 1)$ if and only if $\mu\geq \mu_{0}$.

Let $f(p, x)$, $f_{1}(p, x)$, $f_{2}(p, x)$ be defined by (2.17)-(2.19) and

3.5 $$ g(x)=\frac{(x^{2}+p_{0})^{2}}{x^{2}}f_{2}(p_{0}, x)=\frac {(x^{2}+p_{0})^{2}}{x^{2}} \psi^{\prime}(x+1)-\frac{2p_{0}}{x^{2}} -\frac{(x^{2}+p_{0})^{2}}{x^{2}(x+p_{0})^{2}}+2. $$

Then elaborated computations lead to

3.6 $$\begin{aligned}& f\bigl(p_{0}, 0^{+}\bigr)=f\bigl(p_{0}, 1^{-}\bigr)=0, \end{aligned}$$

3.7 $$\begin{aligned}& f_{1}\bigl(p_{0}, 0^{+}\bigr)= \frac{1-\gamma-\gamma^{2}}{\gamma}>0, \qquad f_{1}\bigl(p_{0}, 1^{-}\bigr)=0, \end{aligned}$$

3.8 $$\begin{aligned}& g\bigl(0^{+}\bigr)=-\infty, \qquad g\bigl(1^{-}\bigr)= \frac{(\pi^{2}-6)p_{0}^{2}+2(\pi ^{2}-12)p_{0}+\pi^{2}}{6}>0, \end{aligned}$$

3.9 $$\begin{aligned}& \begin{aligned}[b] g^{\prime}(x)={}&\frac{(x^{2}+p_{0})^{2}\psi^{\prime\prime }(x+1)}{x^{2}}-\frac{2(p_{0}^{2}-x^{4})\psi^{\prime }(x+1)}{x^{3}} \\ &-\frac{2p_{0} [x^{4}-4x^{3}-6p_{0}x^{2}-2p_{0}(3p_{0}+1)x-(2p_{0}+1)p_{0}^{2} ]}{(x+p_{0})^{3}x^{3}}. \end{aligned} \end{aligned}$$

It follows from the second inequality in (2.1) and the first inequality in (2.2) together with (3.9) that

$$\begin{aligned} g^{\prime}(x)\geq{}&{-}\frac{(x^{2}+p_{0})^{2}}{x^{2}(x+1/2)^{2}}-\frac {2(p_{0}^{2}-x^{4})}{(x+1/2)x^{3}} \\ &-\frac{2p_{0} [x^{4}-4x^{3}-6p_{0}x^{2}-2p_{0}(3p_{0}+1)x-(2p_{0}+1)p_{0}^{2} ]}{(x+p_{0})^{3}x^{3}} \\ ={}&\frac{2}{x^{3}(2x+1)^{2}(x+p_{0})^{3}}g_{1}(x), \end{aligned}$$

where

$$\begin{aligned} g_{1}(x)={}&2x^{8}+2(3p_{0}+1)x^{7}+2p_{0}(3p_{0}-1)x^{6}+2p_{0} \bigl(p_{0}^{2}-3p_{0}+6\bigr)x^{5} \\ &-p_{0}\bigl(10p_{0}^{2}-18p_{0}-15 \bigr)x^{4}-2p_{0}(p_{0}+2) \bigl(2p_{0}^{2}-7p_{0}-1 \bigr)x^{3} \\ &-2p_{0}^{2}\bigl(5p_{0}^{2}-11p_{0}-7 \bigr)x^{2}-2p_{0}^{2}(p_{0}+1) \bigl(3p_{0}^{2}-4p_{0}-1\bigr)x \\ &-p_{0}^{3}\bigl(2p_{0}^{2}-2p_{0}-1 \bigr). \end{aligned}$$

It is easy to verify that all the coefficients of the polynomial $g_{1}(x)$ are positive, which implies that $g(x)$ is strictly increasing on $(0, 1)$, then from (3.5) and (3.8) we know that there exists $\eta\in(0, 1)$ such that the function $f_{1}(p_{0}, x)$ is strictly decreasing on $(0, \eta)$ and strictly increasing on $(\eta, 1)$.

It follows from (2.18) and (3.7) together with the piecewise monotonicity of the function $f_{1}(p_{0}, x)$ on the interval $(0, 1)$ that there exists $x_{0}\in(0, 1)$ such that $f(p_{0}, x)$ is strictly increasing on $(0, x_{0})$ and strictly decreasing on $(x_{0}, 1)$ and $x_{0}$ is the unique solution of equation (3.4) on the interval $(0, 1)$.

Therefore, the desired results follow easily from (2.17), (3.3), (3.6) and the piecewise monotonicity of the function $f(p_{0}, x)$ on the interval $(0, 1)$ together with the fact that the function $p\rightarrow(x^{2}+p)/(x+p)$ is strictly increasing.

Numerical computations show that $x_{0}=0.346\ldots$ and $\mu_{0}=(x_{0}+p_{0})\Gamma(x_{0}+1)/(x^{2}_{0}+p_{0})=1.027\ldots$ . □

### Theorem 3.2

*The inequality*

$$ \Gamma(x+1)>\frac{x^{2}+\frac{1}{\gamma}}{x+\frac{1}{\gamma}} $$

*holds for all* $x\in(0, x^{\ast})$, *and its reverse inequality*

$$ \Gamma(x+1)< \frac{x^{2}+\frac{1}{\gamma}}{x+\frac{1}{\gamma}} $$

*holds for all* $x\in(x^{\ast}, 1)$, *where* $x^{\ast}=0.385\ldots$ *is the unique solution of the equation*

$$ \Gamma(x+1)-\frac{x^{2}+\frac{1}{\gamma}}{x+\frac{1}{\gamma}}=0 $$

*on the interval* $(0, 1)$.

### Proof

Let $f(p, x)$, $f_{1}(p, x)$ and $f_{2}(p, x)$ be, respectively, defined by (2.17), (2.18) and (2.19). Then simple computations lead to

3.10 $$\begin{aligned}& f \biggl(\frac{1}{\gamma}, 0^{+} \biggr)=f \biggl( \frac{1}{\gamma}, 1^{-} \biggr)=0, \end{aligned}$$

3.11 $$\begin{aligned}& f_{1} \biggl(\frac{1}{\gamma}, 0^{+} \biggr)=0, \qquad f_{1} \biggl(\frac {1}{\gamma}, 1^{-} \biggr)= \frac{1-\gamma-\gamma^{2}}{1+\gamma}>0. \end{aligned}$$

From Lemma 2.11 and $1/\gamma=1.732\ldots\in[8/5, 9/5]$ we know that there exist $\eta_{1}(1/\gamma), \eta_{2}(1/\gamma)\in(0, 1)$ with $\eta_{1}(1/\gamma)<\eta_{2}(1/\gamma)$ such that $f_{1}(1/\gamma, x)$ is strictly increasing on $(0, \eta_{1}(1/\gamma))\cup( \eta _{2}(1/\gamma), 1)$ and strictly decreasing on $(\eta_{1}(1/\gamma), \eta_{2}(1/\gamma))$. We claim that

3.12 $$ f_{1}\bigl(1/\gamma, \eta_{2}(1/\gamma)\bigr)< 0. $$

Indeed, if $f_{1}(1/\gamma, \eta_{2}(1/\gamma))\geq0$, then the piecewise monotonicity of the function $f_{1}(1/\gamma, x)$ on the interval $(0, 1)$ and (3.11) lead to the conclusion that $f(1/\gamma, x)$ is strictly increasing on $(0, 1)$, which contradicts (3.10).

It follows from (3.11) and (3.12) together with the piecewise monotonicity of the function $f_{1}(1/\gamma, x)$ on the interval $(0, 1)$ that there exist $\eta ^{\ast}_{1}(1/\gamma)\in(\eta_{1}(1/\gamma), \eta_{2}(1/\gamma))$ and $\eta^{\ast}_{2}(1/\gamma)\in(\eta_{2}(1/\gamma), 1)$ such that $f(1/\gamma, x)$ is strictly increasing on $(0, \eta^{\ast}_{1}(1/\gamma ))\cup(\eta^{\ast}_{2}(1/\gamma), 1)$ and strictly decreasing on $( \eta ^{\ast}_{1}(1/\gamma), \eta^{\ast}_{2}(1/\gamma))$.

Therefore, Theorem 3.2 follows easily from (2.17) and (3.10) together with the piecewise monotonicity of $f(1/\gamma, x)$ on $(0, 1)$. Numerical computations show that $x^{\ast}=0.385\ldots$ . □

### Theorem 3.3

*The double inequality*

3.13 $$ \frac{\lambda ({x^{2}+\frac{9}{5}} )}{x+\frac{9}{5}}\leq\Gamma (x+1)< \frac{{x^{2}+\frac{9}{5}}}{x+\frac{9}{5}} $$

*holds for all* $x\in(0, 1)$ *with the best possible constant*

3.14 $$ \lambda=\frac{(5\tau_{0}+9)\Gamma(\tau_{0}+1)}{5{\tau _{0}}^{2}+9}=0.991\ldots, $$

*where* $\tau_{0}=0.719\ldots$ *is the unique solution of the equation*

3.15 $$ \psi(x+1)-\frac{2x}{x^{2}+\frac{9}{5}}+\frac{1}{x+\frac{9}{5}}=0 $$

*on the interval* $(0, 1)$.

### Proof

Let $f(p, x)$, $f_{1}(p, x)$ and $f_{2}(p, x)$ be, respectively, defined by (2.17), (2.18) and (2.19). Then simple computations lead to

3.16 $$\begin{aligned}& f \biggl(\frac{9}{5}, 0^{+} \biggr)=f \biggl( \frac{9}{5}, 1^{-} \biggr)=0, \end{aligned}$$

3.17 $$\begin{aligned}& f_{1} \biggl(\frac{9}{5}, 0^{+} \biggr)= \frac{5}{9}-\gamma< 0, \qquad f_{1} \biggl(\frac{9}{5}, 1^{-} \biggr)=\frac{9}{14}-\gamma>0. \end{aligned}$$

It follows from Lemma 2.11 that there exist $\eta_{1}(9/5), \eta _{2}(9/5)\in(0, 1)$ with $\eta_{1}(9/5)<\eta_{2}(9/5)$ such that $f_{2}(9/5, x)>0$ for $x\in(0, \eta_{1}(9/5))\cup(\eta_{2}(9/5), 1)$ and $f_{2}(9/5, x)<0$ for $x\in(\eta_{1}(9/5), \eta_{2}(9/5))$, and $f_{1}(9/5, x)$ is strictly increasing on $(0, \eta_{1}(9/5))\cup(\eta_{2}(9/5), 1)$ and strictly decreasing on $(\eta_{1}(9/5), \eta_{2}(9/5))$. Then Lemmas 2.7-2.9 lead to the conclusion that $\eta_{1}(9/5)\in(7/50, 1/3)$ and

3.18 $$ f_{1}\bigl(9/5, \eta_{1}(9/5)\bigr)< 0. $$

From (2.18), (3.17), (3.18) and the piecewise monotonicity of $f_{1}(9/5, x)$ on $(0, 1)$ we clearly see that there exists $\tau_{0}$ such that $\tau_{0}$ is the unique solution of equation (3.15) on the interval $(0, 1)$, and $f(9/5, x)$ is strictly decreasing on $(0, \tau _{0})$ and strictly increasing on $(\tau_{0}, 1)$.

Equation (3.16) and the piecewise monotonicity of the function $f(9/5, x)$ on the interval $(0, 1)$ lead to the conclusion that

3.19 $$ f(9/5, \tau_{0})\leq f(9/5, x)< 0 $$

for all $x\in(0, 1)$.

Therefore, inequality (3.13) holds for all $x\in(0, 1)$ follows from (2.17) and (3.19). We clearly see that the parameter *λ* given by (3.14) is the best possible constant such that the first inequality in (3.13) holds for all $x\in(0, 1)$. Numerical computations show that $\tau _{0}=0.719\ldots$ and $\lambda=0.991\ldots$ . □

### Remark 3.4

From Theorems 3.1 and 3.3 we clearly see that the double inequality

3.20 $$ \frac{x^{2}+p_{0}}{x+p_{0}}< \Gamma(x+1)< \frac{x^{2}+p_{1}}{x+p_{1}} $$

holds for all $x\in(0, 1)$ with $p_{0}=\gamma/(1-\gamma)=1.365\ldots$ and $p_{1}=9/5$, the constant $p_{0}$ appears to be the best possible, but this is not true for $p_{1}$, and a slightly smaller value for $p_{1}$ is possible. Unfortunately, we cannot find the best possible constant $p_{1}$ in the article; we leave this as an open problem for the reader.

### Remark 3.5

From the monotonicity of the function $p\mapsto(x^{2}+p)/(x+p)$ we clearly see that both the upper and lower bounds for $\Gamma(x+1)$ given in (3.20) are better than that given in (1.3), and the first (second) inequality in Theorem 3.2 is the improvement of the first (second) inequality in (1.3) for $x\in(0, x^{\ast})$ $(x\in(x^{\ast}, 1))$, where $x^{\ast}=0.385\ldots$ is given by Theorem 3.2.

### Remark 3.6

From Lemma 2.6, $\gamma+\gamma^{2}<1$, $1/\gamma>1$, $\gamma/(1-\gamma )>1$ and $(x^{2}+1)/(x+1)<1$ for $x\in(0, 1)$ one has

$$\begin{gathered} \frac{x^{2}+\frac{\gamma}{1-\gamma}}{x+\frac{\gamma}{1-\gamma}}> \biggl(\frac{x^{2}+1}{x+1} \biggr)^{2(1-\gamma)}, \\\biggl(\frac{x^{2}+1}{x+1} \biggr)^{\gamma}>\frac{x^{2}+\frac{1}{\gamma }}{x+\frac{1}{\gamma}}> \biggl( \frac{x^{2}+1}{x+1} \biggr)^{2\gamma /(1+\gamma)} > \biggl(\frac{x^{2}+1}{x+1} \biggr)^{2(1-\gamma)}. \end{gathered}$$

Therefore, the lower bound for $\Gamma(x+1)$ given in (3.20) is better than that given in (1.4), the first inequality in Theorem 3.2 is an improvement of the first inequality in (1.4) for $x\in(0, x^{\ast})$ and the second inequality in Theorem 3.2 is an improvement of the second inequality in (1.4) for $x\in(x^{\ast}, 1)$, where $x^{\ast }=0.385\ldots$ is given by Theorem 3.2.

### Remark 3.7

It is not difficult to verify that

$$\begin{gathered} \min_{x\in(0,1)} \biggl(\frac{x^{2}+1}{x+1} \biggr)^{\gamma}= \bigl[2(\sqrt {2}-1)\bigr]^{\gamma}=0.897\ldots, \\\min_{x\in(0,1)} \bigl(2^{1-x}x^{x} \bigr)=2e^{-2/e}=0.958\ldots, \\\frac{x^{2}+\frac{9}{5}}{x+\frac{9}{5}}< 0.89 \end{gathered}$$

for $x\in(0.44, 0.45)$ and

$$ \frac{x^{2}+\frac{9}{5}}{x+\frac{9}{5}}< 0.95 $$

for $x\in(\theta_{0}, \theta_{1})$, where $\theta_{0}=(0.95-\sqrt {0.5425})/2=0.106\ldots$ and $\theta_{1}=(0.95+\sqrt {0.5425})/2=0.843\ldots$ . Therefore, the upper bound $(x^{2}+9/5)/(x+9/5)$ for $\Gamma(x+1)$ given in (3.20) is better than that given in (1.4) for $x\in(0.44, 0.45)$, and it is also better than that given in (1.2) for $x\in(\theta_{0}, \theta_{1})$.

### Remark 3.8

Let

$$ L_{3}(x)= \biggl(\frac{1}{2}+\sqrt{\frac{1}{4}+x} \biggr)^{1-x}x^{x}, \qquad L_{4}(x)= \frac{x^{2}+\frac{\gamma}{1-\gamma}}{x+\frac{\gamma }{1-\gamma}}. $$

Then numerical computations show that

$$\begin{gathered} L_{3}(1/8)=0.846\ldots< L_{4}(1/8)=0.926\ldots, \\L_{3}(1/4)=0.814\ldots< L_{4}(1/4)=0.883\ldots, \\L_{3}(3/8)=0.811\ldots< L_{3}(3/8)=0.865\ldots, \\L_{3}(1/2)=0.826\ldots< L_{4}(1/2)=0.865\ldots, \\L_{3}(5/8)=0.794\ldots< L_{4}(5/8)=0.882\ldots, \\L_{3}(3/4)=0.891\ldots< L_{4}(3/4)=0.911\ldots, \\L_{3}(7/8)=0.913\ldots< L_{4}(7/8)=0.951\ldots. \end{gathered}$$

Therefore, there exists $\delta\in(0, 1/8)$ such that the lower bound for $\Gamma(x+1)$ given in (3.20) is better than that given in (1.2) for $x\in(\delta, 1/8+\delta)\cup(1/4-\delta, 1/4+\delta)\cup (3/8-\delta, 3/8+\delta)\cup(1/2-\delta, 1/2+\delta)\cup(5/8-\delta, 5/8+\delta)\cup(3/4-\delta, 3/4+\delta)\cup(7/8-\delta, 7/8+\delta)$.

## Results and discussion

In this paper, we provide the accurate bounds for the classical gamma function in terms of very simple rational functions, which can be used to estimate the value of the gamma function in the area of engineering and technology.

## Conclusion

In the article, we present several very simple and practical rational bounds for the gamma function, which can be regarded as a simple estimation of the value of the gamma function. The given results are improvements of some well-known results.
